# Placental Gene Transcript Proportions are Altered in the Presence of *In Utero* Arsenic and Cadmium Exposures, Genetic Variants, and Birth Weight Differences

**DOI:** 10.3389/fgene.2022.865449

**Published:** 2022-05-13

**Authors:** Maya A. Deyssenroth, Shouneng Peng, Ke Hao, Carmen J. Marsit, Jia Chen

**Affiliations:** ^1^ Department of Environmental Health Sciences, Mailman School of Public Health, Columbia University, New York, NY, United States; ^2^ Institute of Genomics and Multiscale Biology, Icahn School of Medicine at Mount Sinai, New York, NY, United States; ^3^ Department of Genetics and Genomic Sciences, Icahn School of Medicine at Mount Sinai, New York, NY, United States; ^4^ Department of Environmental Health, Rollins School of Public Health, Emory University, Atlanta, GA, United States; ^5^ Department of Environmental Medicine and Public Health, Icahn School of Medicine at Mount Sinai, New York, NY, United States

**Keywords:** placenta, arsenic, cadmium, birth weight, sQTL, DTU

## Abstract

**Background:** *In utero* arsenic and cadmium exposures are linked with reduced birth weight as well as alterations in placental molecular features. However, studies thus far have focused on summarizing transcriptional activity at the gene level and do not capture transcript specification, an important resource during fetal development to enable adaptive responses to the rapidly changing *in utero* physiological conditions. In this study, we conducted a genome-wide analysis of the placental transcriptome to evaluate the role of differential transcript usage (DTU) as a potential marker of *in utero* arsenic and cadmium exposure and fetal growth restriction.

**Methods:** Transcriptome-wide RNA sequencing was performed in placenta samples from the Rhode Island Child Health Study (RICHS, *n* = 199). Arsenic and cadmium levels were measured in maternal toenails using ICP-MS. Differential transcript usage (DTU) contrasting small (SGA) and appropriate (AGA) for gestational age infants as well as above vs. below median exposure to arsenic and cadmium were assessed using the DRIMSeq R package. Genetic variants that influence transcript usage were determined using the sQTLseeker R package.

**Results:** We identified 82 genes demonstrating DTU in association with SGA status at an FDR <0.05. Among these, one gene, *ORMDL1*, also demonstrated DTU in association with arsenic exposure, and fifteen genes (*CSNK1E*, *GBA*, *LAMTOR4*, *MORF4L1*, *PIGO*, *PSG1*, *PSG3*, *PTMA*, *RBMS1*, *SLC38A2*, *SMAD4*, *SPCS2*, *TUBA1B*, *UBE2A*, *YIPF5*) demonstrated DTU in association with cadmium exposure. In addition to cadmium exposure and SGA status, proportions of the *LAMTOR4* transcript ENST00000474141.5 also differed by genetic variants (rs10231604, rs12878, and rs3736591), suggesting a pathway by which an *in utero* exposure and genetic variants converge to impact fetal growth through perturbations of placental processes.

**Discussion:** We report the first genome-wide characterization of placental transcript usage and associations with intrauterine metal exposure and fetal growth restriction. These results highlight the utility of interrogating the transcriptome at finer-scale transcript-level resolution to identify novel placental biomarkers of exposure-induced outcomes.

## Introduction

Fetal growth is susceptible to varying *in utero* conditions and environmental exposures. In particular, trace toxic metals and metalloids are linked to reduced birth weight as well as health effects in childhood (Chung et al., 2015; Valeri et al., 2017; Wang G. et al., 2021; McRae et al., 2022). Specifically, several reports link reductions in birth weight to *in utero* levels of arsenic (Xu et al., 2011; Deyssenroth et al., 2018) and cadmium (Llanos and Ronco, 2009; Shirai et al., 2010; Luo et al., 2017; Sabra et al., 2017; Deyssenroth et al., 2018; Freire et al., 2019; Punshon et al., 2019). A growing body of evidence links placental programming disruptions in the pathway between exposure to these metals and fetal growth restriction (Punshon et al., 2019). For example, we and others have shown that gestational exposure to arsenic and cadmium is associated with being born small for gestational age (SGA), and levels of these metals covary with the altered expression of placental gene networks implicated with SGA status. (Deyssenroth et al., 2018).

Variations in transcripts that map to a gene, including alternative start and stop codons and skipped exons, are realized through alternative splicing and other transcriptional process. While the overall function of a protein is generally conserved across transcripts, the preferential expression of specific transcripts can inform where the encoded protein is localized within a cell, whether it is exported, its enzymatic activity, and binding capacity to interact with other proteins and nucleic acid (Kelemen et al., 2013). Modulating expression at the level of transcripts can, thereby, increase the diversity in protein function to dynamically respond to developmental stage-specific needs and environmental cues. A homeostasis in transcript-proportions is generally maintained under normal conditions, and a shift in this balance has implications for health (Kim et al., 2018).

Perinatal studies evaluating transcriptomic markers of exposure-induced birth outcomes typically evaluate gene expression (i.e., total transcript abundance for each gene) differences to identify loci of interest. While summarizing transcriptome-wide data to gene-level resolution is a convenient and useful metric to conduct differential expression analysis, it may also obscure the true underlying variability in transcriptomic regulation. Indeed, altered proportions of transcripts across conditions can result in no detectable overall differences in gene expression. Current advances in sequencing technology allow for more fine-scaled interrogations to capture transcript-level differences in expression. These include describing changes in proportions among transcripts within a gene, also referred to as differential transcript usage (DTU).

Most studies to date have linked changes in gene transcript-level expression to cancer (Zhang and Manley, 2013) and neurodegenerative outcomes (Dredge et al., 2001). In addition, *in vitro* and *in vivo* studies have also linked environmental stressors, including alcohol (Sariyer et al., 2017), methyl-mercury (Li et al., 2018) and social interaction, as well as genetic variants as sources of transcript expression profile perturbations. However, the relevance of alterations in transcript proportions as markers of environmental exposures and birth outcomes is understudied in population studies. This is despite the relevance of transcript-specification for the adaptive progression through fetal development and its potential to reflect disruptions in this process. This study seeks to evaluate the association between placental transcript proportions, arsenic and cadmium exposure and birth weight in a birth cohort study.

## Methods

### Study Population

Mother-infant pairs were recruited at Women and Infants Hospital in Providence, Rhode Island as part of the Rhode Island Child Health Study (*n* = 841). Participants who met eligibility criteria included women with singleton pregnancies who were at least 18 years of age, delivered at term (≥37 weeks gestation) and did not experience major pregnancy or offspring complications (e.g., chromosomal abnormalities, congenital defects). The study population was oversampled for small for gestational age (SGA, < 10% Fenton growth curve) and large for gestational age (LGA, > 90% Fenton growth curve) infants. Appropriate for gestational age (AGA) infants were concurrently enrolled and matched for gestational and maternal age. Informed consent was obtained from all participants, and the study was reviewed and approved by the Internal Review Boards at Woman and Infants Hospital and Emory University. The current study is restricted to participants with available placental RNA seq data and no reported maternal smoke exposure during pregnancy (*n* = 196). The birth weight analysis focused on comparing SGA (*n* = 30) and AGA (*n* = 112) infants. Arsenic and cadmium measurements were available for 171 participants.

### Trace Metal Analyses

ICP-MS was performed at the Dartmouth Trace Metal laboratory to determine metal levels across a panel of 19 metals measured in toenail clippings obtained from RICHS mothers and infants following hospital discharge as previously described (Everson et al., 2016; Punshon et al., 2016; Everson et al., 2017; Deyssenroth et al., 2018). Briefly, toenail clippings were received from participants, on average, within 2.8 months postpartum. Study samples were cleaned, microwave digested, and analyzed alongside certified reference materials *via* ICP-MS at the Dartmouth Trace Element Analysis Core following guidelines outlined in EPA 6020A. Metal measurements are expressed as microgram (μG) metal per gram toenail. Values falling below the sample-specific limit of detection (LOD) were replaced with the $\frac{LOD}{\surd 2}$ . The current study focuses on arsenic and cadmium exposure levels assessed in maternal toenails based on our previous findings linking these measurements to fetal growth restriction in this population (Deyssenroth et al., 2018).

### Genomic Analyses

Placenta collection, placental transcriptome and SNP genotyping data acquisition were performed as previously described (Peng et al., 2017; Clarkson-Townsend et al., 2020). Briefly, placenta tissue was biopsied 2 cm from the umbilical cord insertion site, free from maternal decidua, within 2 h of delivery. Biopsies were placed in RNALater and maintained at 4°C for 72 h prior to storage at −80°C. RNA was extracted using the RNeasy mini kit (Qiagen, Valencia, CA) following manufacturer’s instructions. Single-end 50 bp RNA sequencing reads were generated using the Illumina HiSeq 2,500 platform. SNP genotyping data was generated using the Illumina MegaEX chip and processed as previously described (Peng et al., 2017).

### Statistical Analysis

Transcript abundance was quantitated based on the GRCh38.12 v28 human reference genome using salmon. (Patro et al., 2017). The total detected counts (# transcripts = 203,027) were restricted to protein-coding genes (# transcripts = 147,015, # genes = 19,989). Our survey was additionally restricted to genes with at least two transcripts to detect differential transcript usage. The data was further filtered to retain transcripts and genes meeting a minimum expression threshold in at least the number of samples of our smallest phenotypic group (i.e., SGA = 30; As and Cd = 85) based on the following criteria: genes with a minimum overall gene expression of 10 estimated counts, transcripts within genes with a minimum transcript expression of 10 estimated counts, and transcripts accounting for a minimum of 10% of total expression for a given gene. The final datasets consisted of 5,660 genes (23,168 transcripts) for the SGA analysis and 5,057 genes (15,998 transcripts) for the arsenic and cadmium analyses. Differential transcript usage (DTU) contrasting SGA and AGA placenta was performed using the DRIMSeq R package (Nowicka and Robinson, 2016). For the metal DTU analysis, samples were dichotomized above and below the median (Arsenic = 0.04 ug/g; Cadmium = 0.01 ug/g). We repeated testing for metal DTU in a subsetted analysis, restricting our contrast to individuals in the 3rd (*n* = 57) vs. 1st tertile (*n* = 58) of exposure. As an additional control on false discovery rate, a posthoc filtering procedure was performed whereby *p*-values were set to one for transcripts with small standard deviations in per-sample proportions. Significant *p*-values were determined using the stageR R package (Van den Berge and Clement, 2021). In this two-stage testing procedure, a screen to identify genes exhibiting differential transcript usage is performed in the first stage, and transcripts participating in the differential transcript usage of those genes are confirmed in the second stage. Gene ontology enrichment analyses were performed using the enrichR (Kuleshov et al., 2016) R package. We profiled genetic polymorphisms associated with differential transcript usage, also known an splice quantitative trait loci (sQTLs), using the sQTLseeker R package (Monlong et al., 2014). The identified sQTLs were further queried for known disease associations reported in the NHGRI-EBI GWAS catalog. SGA differential transcript usage analyses were adjusted for maternal race/ethnicity and sex. Arsenic and cadmium differential transcript usage analyses were additionally adjusted for metal analysis batch. All analyses were conducted using R 4.1.1. The code implemented for the presented differential transcript usage analysis is available here: https://github.com/Deyssenroth-Lab/RICHS_DTU/tree/master.

## Results

The demographic characteristics contrasting SGA and AGA infants in our study are shown in Table 1. The same demographic characteristics contrasting infants above and below median arsenic and cadmium levels are shown in Supplementary Tables S1, S2. SGA and AGA infants differed by maternal race/ethnicity (greater proportion of non-white mothers among SGA infants compared to AGA infants). Infants above vs. below the median fetal cadmium exposure differed by sex (greater proportion of female infants in group with above median levels of cadmium exposure) and ICP-MS batch (Supplementary Table S2).

**TABLE 1:** Study participant characteristics (*n* = 142).

	AGA (*n* = 112)	SGA (*n* = 30)	*p*-value
	*n* (*%*)	*n* (*%*)	
Infant sex (Female)	57 (50.9)	20 (66.7)	0.18
Maternal race/ethnicity			<0.01
White	91 (81.2)	17 (56.7)	
Black	2 (1.8)	6 (20.0)	
Other	19 (17.0)	7 (23.3)	
Delivery mode (Vaginal)	65 (58.0)	18 (60.0)	1.00
Parity (nulliparous)	40 (36.0)	16 (53.3)	0.13
	***Mean* (*SD*)**	***Mean* (*SD*)**	
Birthweight (grams)	3,436.8 (388.9)	2,581.2 (288.1)	<0.01
Gestational age (weeks)	39.1 (0.9)	38.9 (1.2)	0.40
Maternal age (years)	31.1 (4.6)	32.5 (5.4)	0.18
Maternal BMI (kg/m^2^)	25.6 (5.9)	26.0 (7.3)	0.72
Arsenic (ug/g)	0.04 (0.03)	0.07 (0.08)	0.02
Cadmium (ug/g)	0.02 (0.02)	0.01 (0.01)	0.67

**FIGURE 1:**
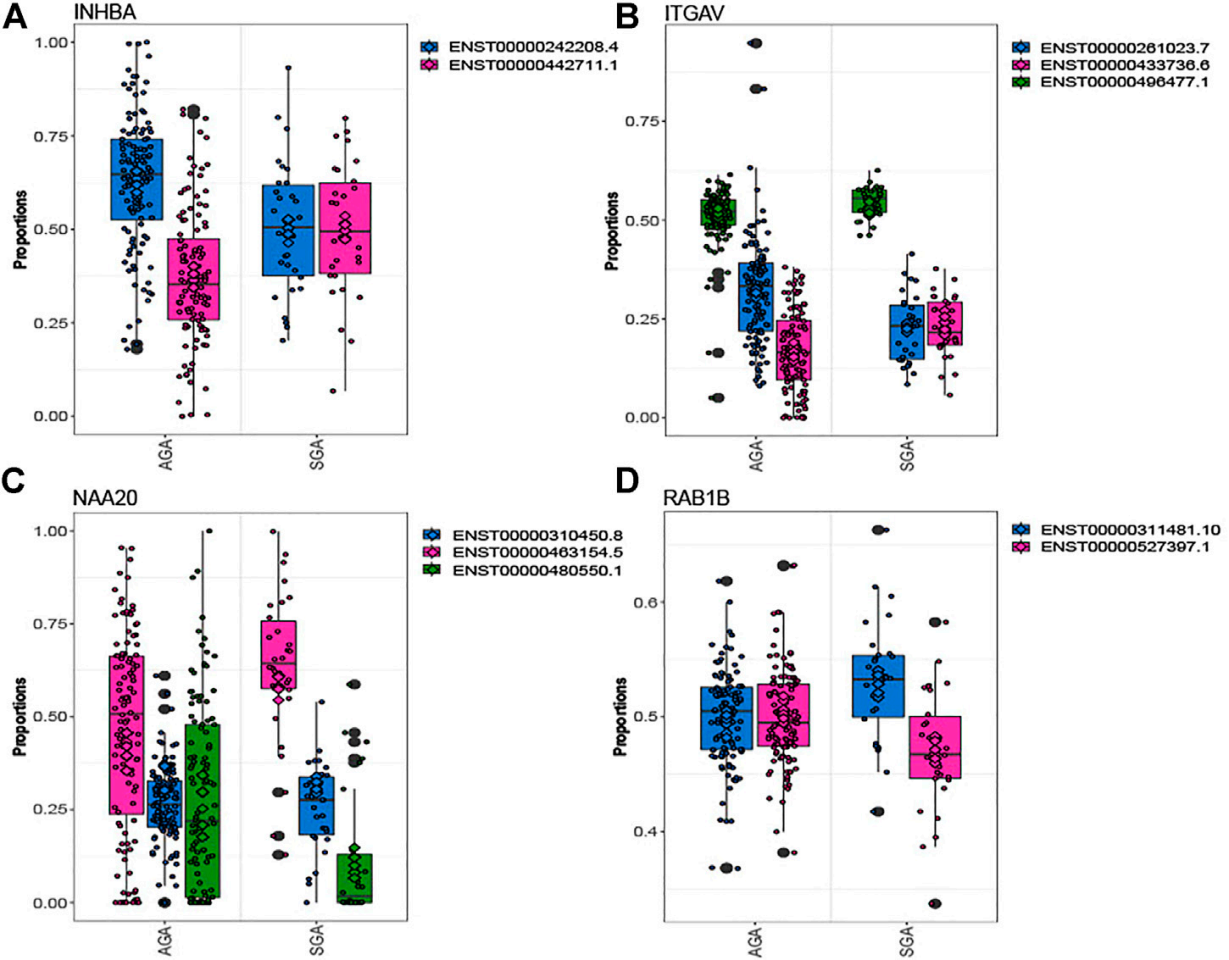
Genes depicting differential transcript usage between SGA and AGA infants. Eighty-two genes demonstrated significant differential transcript usage. Shown are examples of four genes, INHBA **(A)**, ITGAV **(B)**, NAA20 **(C)**, and RAB1B **(D)**. The x-axis indicates the proportion of total measured gene expression captured by individual transcripts.

Out of 5,660 tested genes, we identified 82 genes that displayed differential transcript usage comparing SGA vs. AGA infants (Supplementary Table S3). Examples of genes demonstrating differential transcript usage are shown in Figure 1. This figure depicts instances where equimolar levels of transcripts are present in one condition, and preferential selection of a specific transcript is apparent in the other condition. For example, equivalent placental levels of *INHBA* (ENST00000242208.4; ENST00000442711.1) and ITGAV (ENST00000261023.7; ENST0000433736.6) transcripts are observed among SGA infants (Figures 1A,B). However, one transcript is preferentially expressed (*INHBA*: ENST00000242208.4; *ITGAV*: ENST00000261023.7) over the other among AGA infants. An opposing trend is observed for *NAA20* and *RAB1B* (Figures 1C,D). Here, equivalent placental levels (*NAA20*: ENST000004663154.5; ENST00000480550.1) and *RAB1B* (ENST00000311481.0; ENST0000527397.1) transcripts are observed among AGA infants, and one placental transcript is preferentially expressed (*NAA20*: ENST000004663154.5; *RAB1B*: ENST00000311481.0) over the other among SGA infants. Gene ontology enrichment analysis indicated an overrepresentation of ubiquitin-related processes among genes displaying differential transcript usage by SGA status (Supplementary Figure S1).

**FIGURE 2:**
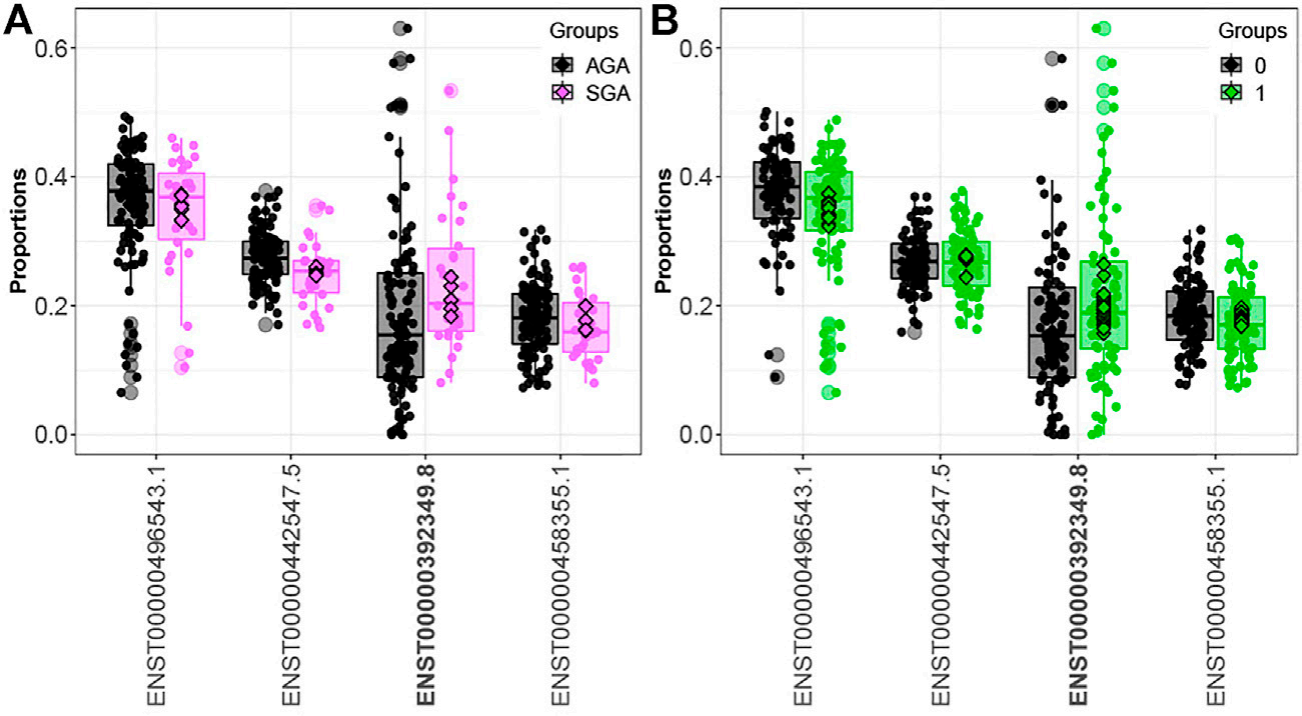
*ORMDL1* differential transcript usage. Elevated expression of the *ORMDL1* transcript ENST00000392349.8 is observed in SGA infants compared to AGA infants **(A)** and infants above vs. below the median fetal exposure to arsenic **(B)**.

Comparing infants above and below the median for fetal arsenic and cadmium exposure, we identified 16 and 160 genes demonstrating differential transcript usage, respectively (Supplementary Tables S4, S5). Similarly, we identified 13 and 155 genes demonstrating differential transcript usage comparing infants in the 3rd vs. 1st tertile of arsenic and cadmium exposure, respectively (Supplementary Tables S6, S7). Overlapping these results with the SGA analysis, one gene (*ORMDL1*) demonstrated differential transcript usage across both the SGA and the dichotomized arsenic analyses. As seen in Figure 2, the direction of the association is consistent across both contrasts (i.e., upregulation of the *ORMDL1* transcript ENST00000392349.8 is observed among both SGA infants and infants above the median for fetal arsenic exposure). However, *ORMDL1* was not among the genes differentially expressed by arsenic exposure when comparing individuals in the 3rd vs. 1st tertile of exposure*.*

**FIGURE 3:**
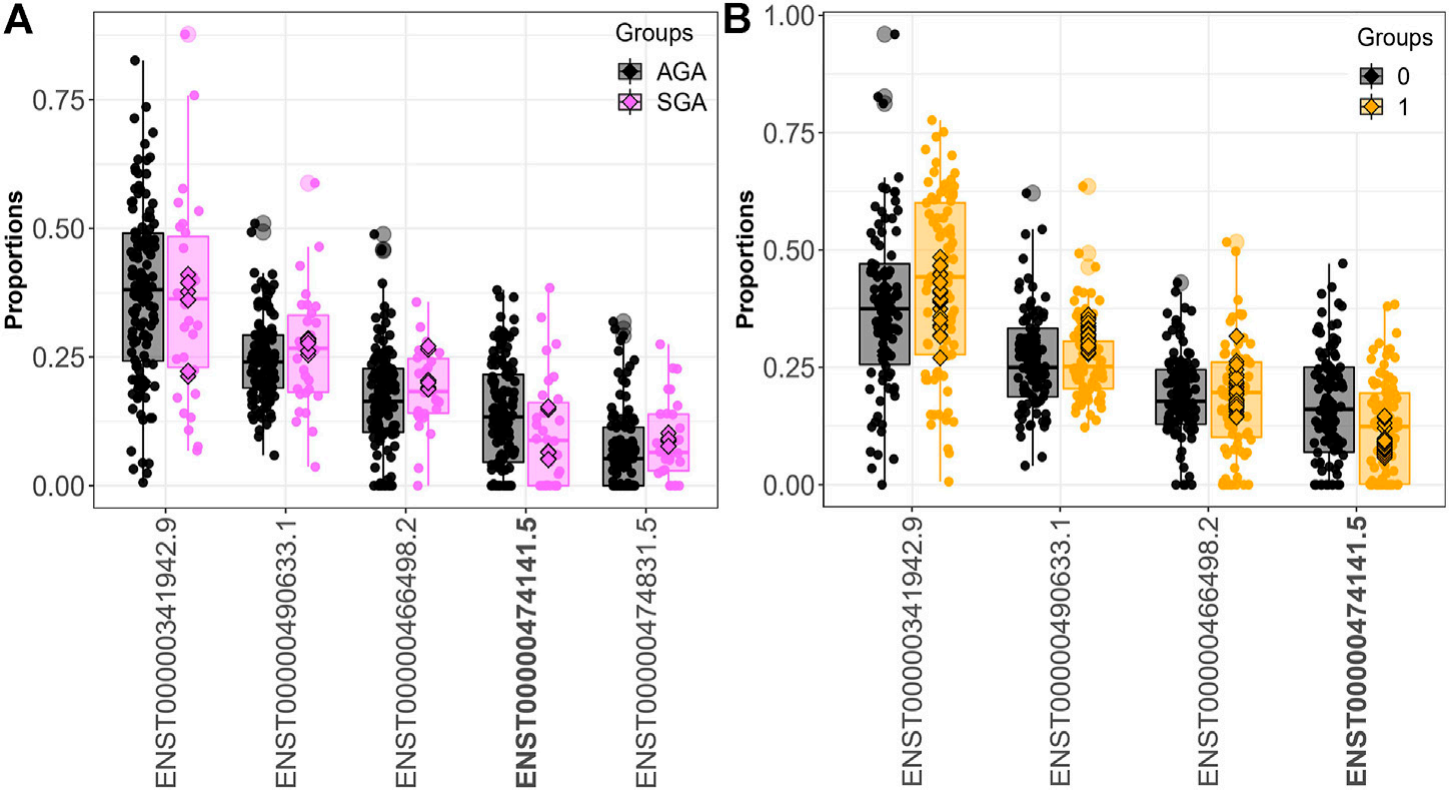
*LAMTOR4* differential transcript usage. Decreased expression of the *LAMTOR4* transcript ENST00000474141.5 is observed in SGA infants compared to AGA infants **(A)** and infants above vs. below the median fetal exposure to **(B)**.

Fifteen genes (*CSNK1E*, *GBA*, *LAMTOR4*, *MORF4L1*, *PIGO*, *PSG1*, *PSG3*, *PTMA*, *RBMS1*, *SLC38A2*, *SMAD4*, *SPCS2*, *TUBA1B*, *UBE2A*, *YIPF5*) demonstrated differential transcript usage across both the SGA and dichotomized cadmium analyses. Out of these fifteen genes, fourteen were also identified when comparing individuals in the 3rd. vs. 1st tertile of exposure (*CSNK1E*, *GBA*, *LAMTOR4*, *PIGO*, *PSG1*, *PSG3*, *PTMA*, *RBMS1*, *SLC38A2*, *SMAD4*, *SPCS2*, *TUBA1B*, *UBE2A*, *YIPF5*). Ten genes (*CSNK1E*, *GBA*, *LAMTOR4*, *PIGO*, *PSG1*, *PSG3*, *PTMA*, *SPCS2*, *TUBA1B*, and *UBE2A*) displayed differential transcript usage of the same transcript and in a consistent direction in association with SGA and above the median fetal cadmium exposure levels. Figure 3 focuses on the overlapping trends for *LAMTOR4*. Here, downregulation of the *LAMTOR4* transcript ENST00000474141.5 is observed among both SGA infants and infants above the median for fetal cadmium exposure.

**FIGURE 4:**
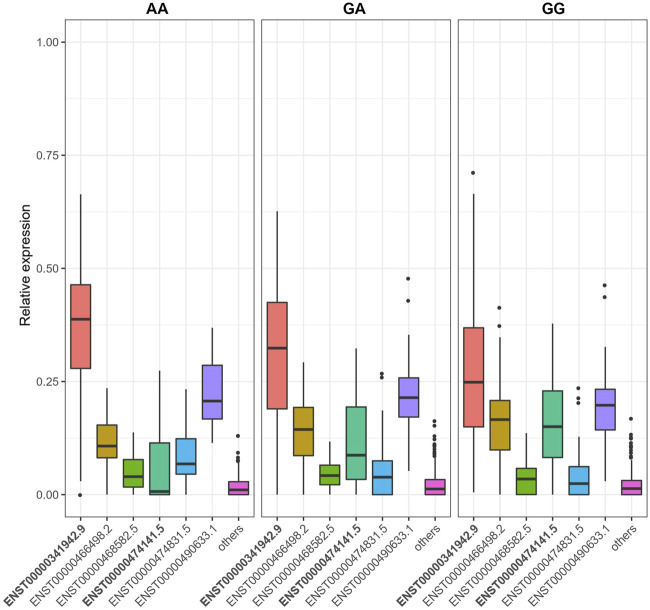
SNP rs10231604 regulates the expression of *LAMTOR4* transcripts. Relative expression of transcript ENST00000341942.9 decreases with increasing dosage of the wildtype allele (G). Relative expression of transcript ENST00000474141.5 increases with increasing dosage of the wildtype allele (G).

We identified 16,528 sQTLs mapping to 1,041 individual genes. Overlaying the sQTL and differential transcript usage findings, we observed one gene, *LAMTOR4*, with a transcript differentially expressed with respect to a SNP variant, SGA status and fetal cadmium exposure. Three SNPs are associated with the expression of *LAMTOR4* transcripts ENST00000474141.5 and ENST00000341942.9. The impact of SNPs rs10231604 [7:100148578], rs12878 [7:100149507], and rs3736591 [7:100153394] are shown in Figure 4, Supplementary Figures S2, S3, respectively. Consistent trends in relative transcript expression patterns are observed for all three SNPs. Increasing dosage of the alternate allele (e.g., rs10231604-A) increases the relative expression of *LAMTOR4* transcript ENST00000341942.9 and decreases the relative expression of *LAMTOR4* transcript ENST00000474141.5. Reductions in the relative expression level of *LAMTOR4* transcript ENST00000474141.5 in the presence of the sQTL variant alleles is consistent with the reductions in relative expression levels of the same transcript observed in association with SGA status and among infants above the median level for fetal cadmium exposure (Figures 3A,B).

**FIGURE 5:**
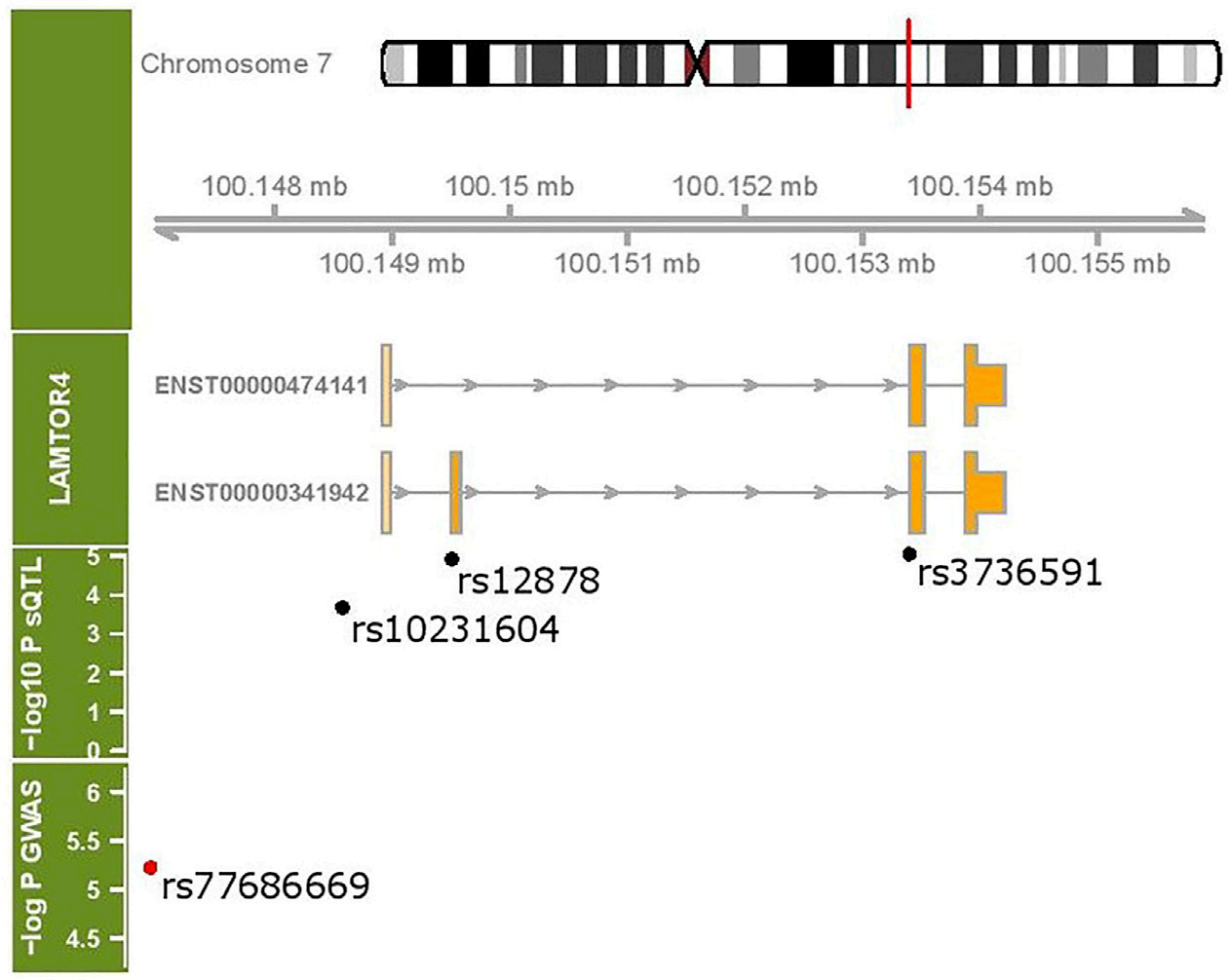
*LAMTOR4* genomic region (chr 7:100148907- 100155944). Two transcripts under sQTL regulation are shown. The location of three sQTL variants influencing the expression of the *LAMTOR4* transcripts are labeled. Shown in red is a GWAS-identified SNP associated with venous thromboembolism.

Figure 5 shows the coding exons for the two transcripts under sQTL regulation as well as the location of the sQTL SNPs with respect to these transcripts. SNP rs10231604 is located upstream of the start codon, SNP rs12878 is located near exon 2, and rs3736591 is located near exon 3. A GWAS-associated SNP (rs77686669) linked with venous thromboembolism in African Americans (Heit et al., 2017) that maps to this region is also shown.

## Discussion

We report here the first study characterizing placental DTU in association with birth weight and *in utero* metal exposure. Several genes we identified in relation to SGA status were previously identified as relevant to placental function based on analyzing overall gene expression differences, including *GBA* (Jebbink et al., 2015), *GCLC* (Hannan et al., 2018), *INHA* (Brew et al., 2016), *INHBA* (Deyssenroth et al., 2018), *ITGAV* (Wang et al., 2019), *NFKBIA* (Mahany et al., 2018), *PSG1* (Whitehead et al., 2013), *RBFOX2* (Goldman- Wohl et al., 2020; Freitag et al., 2021), *SLC38A2* (Vaughan et al., 2021), and *SPIRE2* (Azar et al., 2021).

Consistent literature support also exists for several of the genes we identified in relation to metal exposure. For example, among our arsenic-responsive regions, cord blood methylation of *CD151* was previously associated with drinking water arsenic exposure in a prospective cohort study in Bangladesh (Kile et al., 2014). In a mouse study, altered lung gene expression of *CLIC5* was observed comparing unexposed mice and mice exposed to 10 ppb arsenic in chow or drinking water over a 5–6 week period (Kozul et al., 2009). In an *in vitro* study, *VAV3* was identified as a trivalent arsenical-responsive gene based on gene expression differences comparing treated and untreated HUC-1 cells (Su et al., 2006).

We also identified placental DTU among known cadmium-responsive genes. For example, differential gene expression based on cadmium exposure was observed in *vitro* studies across human breast cancer cell lines (*PECAM1*, *DAB2*, *PGK1*)^23^ (Lubovac-Pilav et al., 2013), human renal epithelial cells (*SETD2*, *TBC1D15, TNPO1*) (Garrett et al., 2013), rat liver cells [*ANGPTL4* (Permenter et al., 2011), *ADM*. (Hsiao and Stapleton, 2009)], and mouse embryonic fibroblast cell lines (*EFEMP1*) (Oldani et al., 2020). Similarly, *in vivo* studies also indicate an impact on gene expression patterns in rat hypothalamus (*PSMA1*, *RPLP0*, *SHC1*) (Saedi et al., 2021), mouse testes (*LYE6*) (Hu et al., 2014), mouse pup heart (*ALDOA*). (Hudson et al., 2019), mouse liver (*PRKCE*) (Jackson et al., 2020), and mouse femur (*EEF1A1*) (Ohba et al., 2007). Our study adds to this body of literature by demonstrating that the expression pattern of these genes is also disrupted due to exposure to these metals in human placenta.

Importantly, we also identified many loci previously unreported in relation to SGA status, arsenic and cadmium exposure in this study. This suggests that surveying the placental transcriptome at transcript-level resolution may reveal an additional layer of insight beyond assessing overall gene expression differences. Indeed, as seen in Figure 1, while the distribution in the proportion of individual transcripts within genes can shift across conditions, this change may not translate to a change in overall gene expression. Our findings, therefore, suggest the relevance of transcript-level expression as a more nuanced placental marker of *in utero* exposures and postnatal outcomes.

We identified a lysosomal signaling gene, *LAMTOR4* (Mu et al., 2017), with altered transcript-level expression comparing SGA and AGA infants, infants above vs. below the median for fetal cadmium exposure, and across SNP alleles (rs10231604, rs12878, rs3736591). Consistent with our finding, these three SNPs are also known to inform *LAMTOR4* splicing patterns across adult tissues (i.e., whole blood, brain, skin adipose tissue, breast, colon, muscle, etc) based on reports by the GTEx biobank. This suggests that genetic regulation of *LAMTOR4* transcript-level expression is a systemic phenomenon that extends beyond the *in utero* period in the placenta.

Specifically, the placental proportion of *LAMTOR4* transcript ENST00000474141.5 is reduced in SGA infants, among infants above the median for fetal cadmium exposure, and infants with greater dosage of the alternate alleles for rs10231604, rs12878 and rs3736591. Interestingly, ENST00000474141.5 does not include exon 2, a feature that distinguishes it from all the other known *LAMTOR4* transcripts. The functional consequence of skipping this exon at the protein level and, more broadly, placental tissue level remains unclear. Additional experimental studies are needed to better understand the impact of transcript selection on placental phenotype.

While the focus of our analysis is on birth weight, a prenatally determined outcome, the perturbations on placental function we identified may also increase susceptibility to health effects experienced later in life. Indeed, SNP variants neighboring the sQTLs we identified are implicated with venous thromboembolism. SNPs in this region also confer genetic risk in developing Alzheimer’s disease (Wang Z. et al., 2021), a disorder that can similarly arise due to vascular pathology. Therefore, our findings suggest that genetic variants and *in utero* environmental exposures (i.e., cadmium) can converge on common transcriptomic targets to disrupt placental programming. These impact growth of the developing fetus prenatally, with potential implications on vascular health extending into the postnatal period.

Several limitations inherent in our study warrant considerations. While a major strength of our study is the availability of *in utero* metal measurements, genotyping and placental RNAseq data, the small sample size of the study precludes more in-depth evaluations. For example, our findings suggest that genetic variants and environmental exposures converge on common targets to disrupt placental programming, suggesting that genetic factors may underly variation in the susceptibility to exposure-induced diseases of prenatal origin. The current study was not powered to formally examine the presence of such effect modification in cadmium-related effects on birth weight due to genotype. Similarly, while we identified genes with concordant transcript usage differences in our birth weight and exposure analyses, this study was not sufficiently powered to formally test whether changes in the expression of specific transcripts mediate the exposure-related effects on birth weight. Finally, RNAseq data was generated on bulk placenta tissue, which entails a mixed composition of cells. The biopsy protocol at the time of tissue collection ensured representative sampling across all study samples. However, we cannot exclude the possibility that differences in cell type abundances across conditions underly the differential expression patterns by birth weight and exposure detected in this study. This impacts the interpretation of the findings from one pointing to direct disruption of protein function at the cellular level to more indirect effects on overall placental function through a shift of cell types. While we cannot specify which effects predominate in the current study, both are biologically relevant.

In summary, we report the first genome-wide characterization of placental transcript usage and associations with intrauterine metal exposure and fetal growth restriction. These results highlight the utility of interrogating the transcriptome at finer-scale transcript-level resolution to identify novel placental biomarkers of exposure-induced outcomes.

## Data Availability

The datasets presented in this study can be found in online repositories. The names of the repository/repositories and accession number(s) can be found below: https://www.ncbi.nlm.nih.gov/gap/cgi-bin/study.cgi?study_id=phs001586.v1.p1.
