# Supplementary figures and images for: Placental Gene Transcript Proportions are Altered in the Presence of In Utero Arsenic and Cadmium Exposures, Genetic Variants, and Birth Weight Differences
Source: Front Genet. 2022 May 13;13:865449. doi: 10.3389/fgene.2022.865449 (PMC9136297; doi:10.3389/fgene.2022.865449)

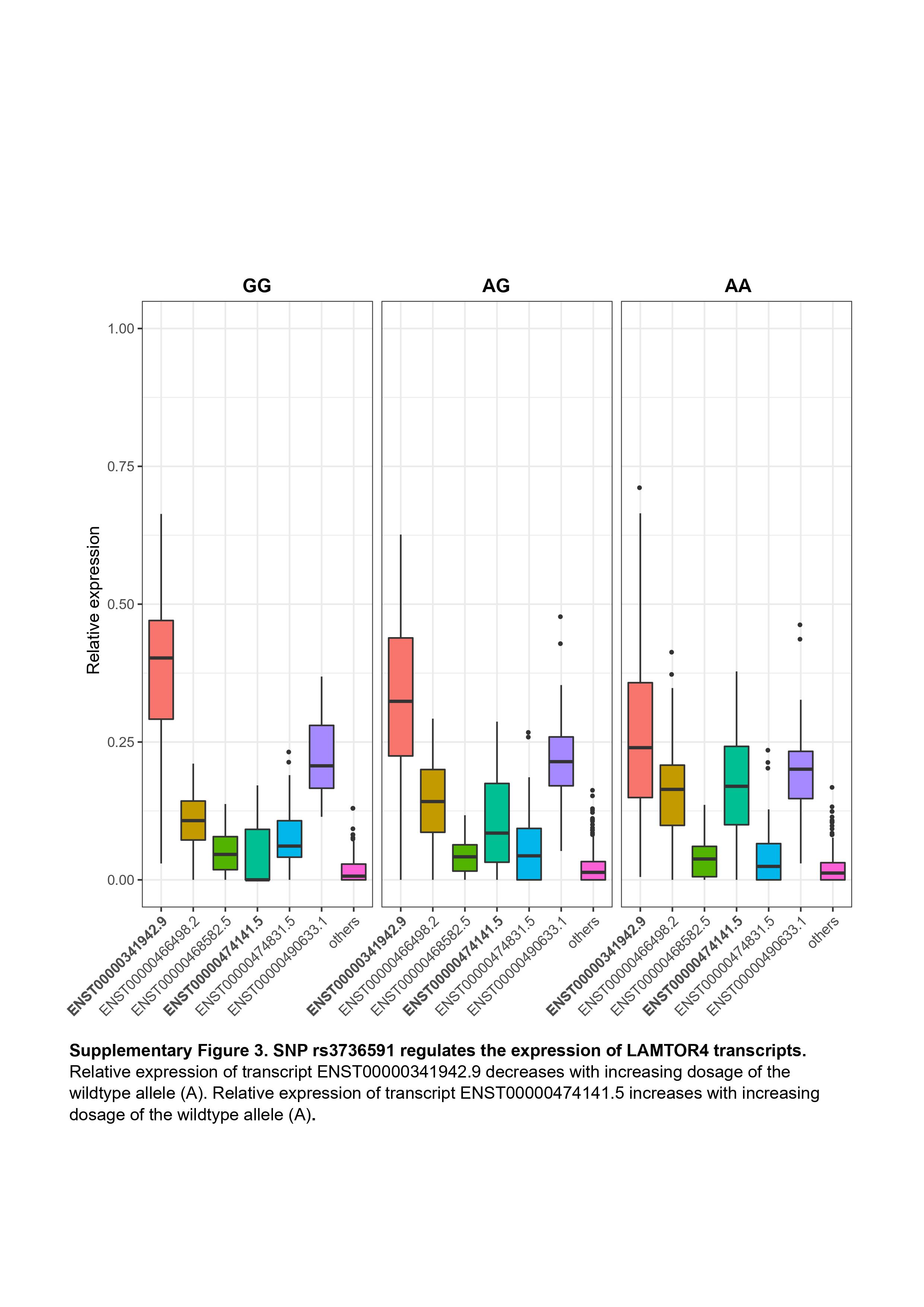

Supplement: Supplementary file 1 [file Image3.jpg]

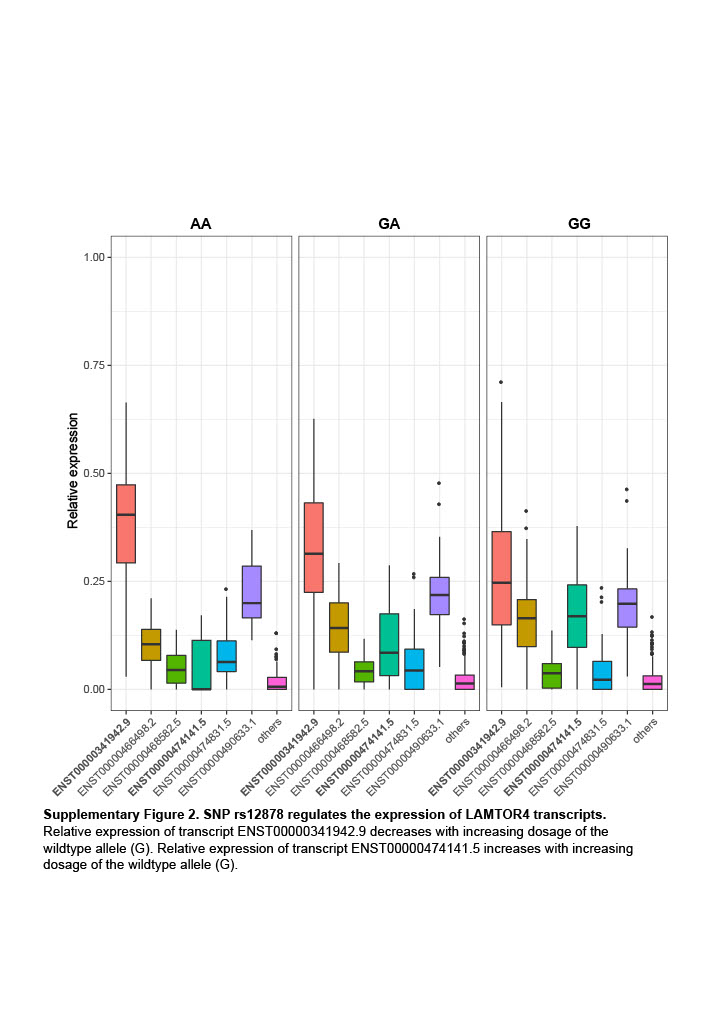

Supplement: Supplementary file 2 [file Image2.jpg]

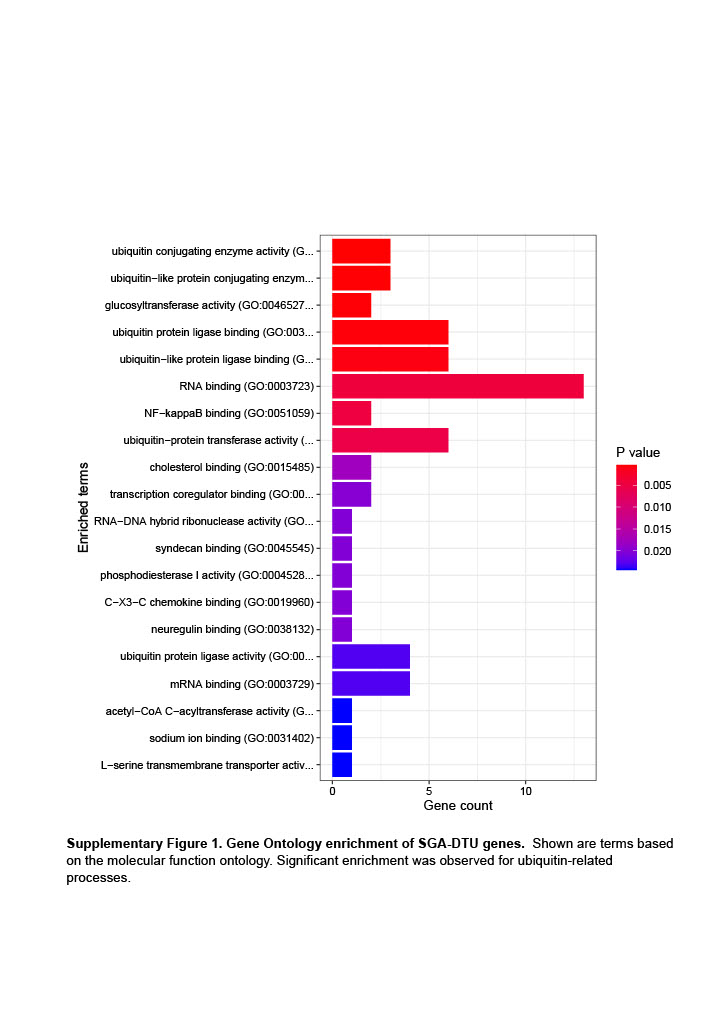

Supplement: Supplementary file 3 [file Image1.jpg]
